# Savior Siblings Might Rescue Fetal Lethality But Not Adult Lymphoma in Irf2bp2-Null Mice

**DOI:** 10.3389/fimmu.2022.868053

**Published:** 2022-07-04

**Authors:** Ragnar O. Vilmundarson, Niloufar Heydarikhorneh, An Duong, Tiffany Ho, Kianoosh Keyhanian, Fariborz Soheili, Hsiao-Huei Chen, Alexandre F. R. Stewart

**Affiliations:** ^1^ Laboratory of Translational Genomics, Ruddy Canadian Cardiovascular Genetics Centre, University of Ottawa Heart Institute, Ottawa, ON, Canada; ^2^ Department of Biochemistry, Microbiology and Immunology, University of Ottawa, Ottawa, ON, Canada; ^3^ Centre for Infection, Immunity and Inflammation, University of Ottawa, Ottawa, ON, Canada; ^4^ Department of Medicine, University of Ottawa, Ottawa, ON, Canada; ^5^ Department of Cellular and Molecular Medicine, University of Ottawa, Ottawa, ON, Canada; ^6^ Brain and Mind Institute, Ottawa Hospital Research Institute, Ottawa, ON, Canada; ^7^ Neuroscience Division, Ottawa Hospital Research Institute, Ottawa, ON, Canada

**Keywords:** germline deletion, myeloid cells, microchimerism, adult lymphoma, transcriptome profiling

## Abstract

Interferon regulatory factor 2 binding protein 2 (Irf2bp2), a co-repressor of Irf2, is required for fetal hepatic erythropoiesis through the expansion of erythromyeloid progenitors. Mice with germline ablation of the entire Irf2bp2 transcript produced no viable Irf2bp2-null pups in first litters. In subsequent litters, fewer than 1/3 of the expected Irf2bp2-null pups were born and half survived to adulthood. As in humans with somatic mutations in IRF2BP2, adult Irf2bp2-null mice developed lymphoma. Transcriptome profiling of liver, heart, and skeletal muscle from Irf2bp2-null adult mice revealed a predominant upregulation of interferon-responsive genes. Of interest, hematopoietic stem cell-enriched transcription factors (Etv6, Fli1, Ikzf1, and Runx1) were also elevated in Irf2bp2-null livers. Intriguingly, Irf2bp2-positive myeloid (but not lymphoid) cells were detected in the livers of adult Irf2bp2-null mice. In female Irf2bp2-null mice, these cells carried a Y chromosome while in male Irf2bp2-null livers, no cells with Barr bodies (inactivated X chromosomes) were detected, indicating that Irf2bp2-positive erythromyeloid cells might be acquired only from male siblings of prior litters by transmaternal microchimerism. These cells likely rescue the deficit in fetal erythropoiesis, but not adult-onset lymphomagenesis, caused by Irfb2p2 ablation.

## Introduction

Interferon-responsive genes are activated by upregulation of interferon regulatory factor 1 (IRF1) that binds to interferon-responsive *cis*-regulatory DNA sequences. Under basal conditions these genes are maintained in a repressed state by competitive binding of the constitutively expressed related factor IRF2 (1). IRF2 owes its repressor function to its interaction with IRF2BP2 (2) that recruits the corepressor NCOR1 (3). IRF2BP2 is one of 3 members of the IRF2BP family of structurally related proteins distinguished by an amino-terminal zinc finger motif and a C-terminal ring finger domain (2). IRF2BP2 also acts as a corepressor of the nuclear factor of activated T cells (NFATC2) (4). On the other hand, we and others have also observed a transactivation function for this protein. We reported that IRF2BP2 acts as a co-activator of the TEAD/VGLL4 complex to promote gene expression in skeletal and cardiac muscle cells (5). More recently, IRF2BP2 was also reported to co-activate transcription regulated by the glucocorticoid and androgen steroid hormone receptors (6). Human IRF2BP2 genetic polymorphisms that reduce IRF2BP2 expression are tied to coronary atherosclerosis (7) and coronary artery calcification (8). Selective ablation of Irf2bp2 in macrophages triggers an inflammatory response and worsens atherosclerosis in mice (7).

Mice with global ablation of Irf2bp2 by a gene trap mutation that inserts a splice acceptor after the first exon die during fetal development (3). The lethal phenotype was tied to deficient fetal erythropoiesis (3), a process that relies on the expansion of an erythromyeloid lineage in the fetal liver (9). These Irf2bp2-deficient mice produce a truncated Irf2bp2 chimeric mRNA that contains the zinc finger motif encoded by the first exon fused to a β-galactosidase/neomycin transcript. The zinc finger motif of Irf2bp2 mediates protein-protein interaction with other Irf2bp family members, whereas the nuclear localization signal of Irf2bp2 is encoded by the second exon (10). Thus, it was unclear to what extent the lethal phenotype of the gene trap mutant was due to loss of functional Irf2bp2 or aberrant function of the chimeric protein.

In humans, rare germline autosomal dominant mutations in IRF2BP2 have been linked to a familial form of common variable immune deficiency disorder (11, 12). On the other hand, somatic mutations of IRF2BP2 are associated with tumors of the lymphoid lineage, suggesting a role of IRF2BP2 loss-of-function in lymphomagenesis. For example, mutations that cause fusions of the IRF2BP2 transcript with the retinoic acid receptor RARA are found in human promyelocytic leukemia (13, 14). Single nucleotide substitutions in the coding sequence of IRF2BP2 mRNA are often detected in patients with primary mediastinal large B cell lymphoma (15, 16) and in T cell lymphoma (17). To date, a mouse model replicating the lymphomagenic phenotype observed in humans with loss-of-function mutations in Irf2bp2 has not been reported.

Lethal anemias can be rescued with transplantation of human leukocyte antigen (HLA)-matched bone marrow from a sibling. If none exists, some parents resort to preimplantation genetic diagnosis of human zygotes after *in vitro* fertilization to select embryos for implantation that are free of the genetic defect causing the anemia yet are HLA-compatible with their child affected by the lethal anemia. After birth, these “savior” siblings provide their bone marrow for transplantation to their affected sibling to replace the defective hematopoietic stem cells (18). Here, we describe a natural process of transmaternal microchimerism in mice where a lethal deficit in fetal erythropoiesis due to loss of Irf2bp2 appears to be rescued by erythromyeloid stem cells that are retained in the mother from male “savior” siblings of prior litters.

## Results

**Figure 1:**
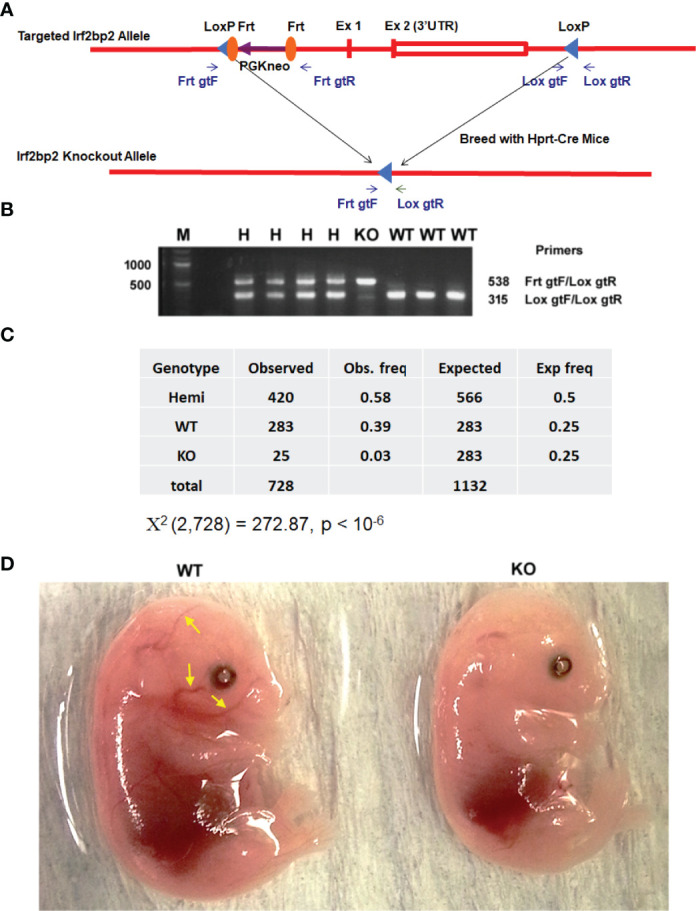
Germline deletion of Irf2bp2. **(A)** Diagram of the construct shows positions of the LoxP sites flanking the Irf2bp2 gene as well as the Frt sites flanking the neomycin selection cassette. **(B)** PCR genotyping of ear biopsy DNA from wild type (WT), hemizygous (H) and Irf2bp2 null (KO) mice. **(C)** Assuming a 1:2:1 Mendelian ratio expected for WT:H:KO genotypes, a non-Mendelian ratio of H and KO progeny was observed. **(D)** Absence of blood-filled cranial vessels (yellow arrows) in an Irf2bp2-null embryo compared to a littermate WT embryo at 15.5 dpc.

We generated mice with loxP sites that bracket the entire Irf2bp2 gene (~6,000 base pairs, including exon 1, exon 2 and the intron) (7). These mice were mated to Hprt1-Cre mice (19) to obtain germline deletion of Irf2bp2 (**Figures 1A, B**). A non-Mendelian ratio of Irf2bp2 null and hemizygous progeny was observed (**Figure 1C**). Over a 9-year period, the occurrence of newborn Irf2bp2-null mice was initially thought to represent rare cases that survived fetal lethality, but further examination revealed that Irf2bp2-null mice only occurred in 2^nd^ and 3^rd^ litters of multiparous dams. No Irf2bp2 null mouse was found in first litters (168) and only a small number of Irf2bp2 null mice were born from 2^nd^ or 3^rd^ litters (25 in 62 litters, ~1/3 of expected) of multiparous dams. Thus, most Irf2bp2 null mice die during development. Half of Irf2bp2 null mice born from 2^nd^ and 3^rd^ litters died within the first few days after birth while the other half survived up to 1 year. To determine why Irf2bp2 null mice die during development, a series of embryos from 9.5 to 18.5 days post-coitum (dpc) was examined in first litters of pregnant dams. Viable null embryos were detected up to 15.5 dpc, but not at 16.5 or 18.5 dpc. Of the only 2 Irf2bp2-null embryos at 15.5 dpc, one was degenerating and the other had a marked reduction in blood-filled vessels (**Figure 1D**, compare littermate WT mouse, yellow arrows), in line with the requirement for Irf2bp2 in fetal liver erythropoiesis (3).

**Figure 2:**
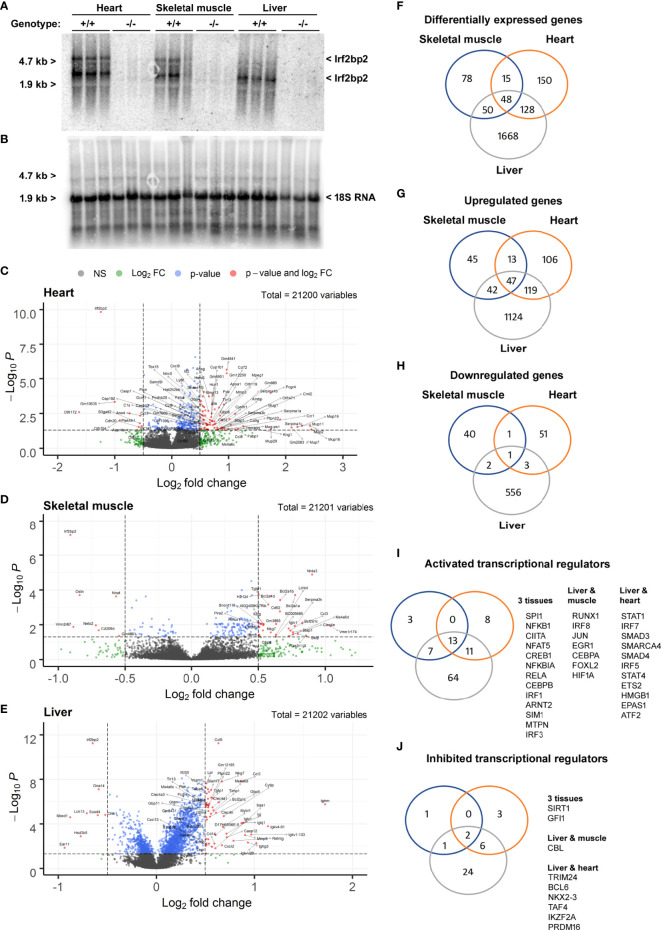
Differentially expressed genes in heart, skeletal muscle, and liver from Irf2bp2-null adult mice. **(A)** Northern blot with total RNA from heart, skeletal muscle and liver of 3 wild type (WT) and 3 Irf2bp2 null (KO) male mice probed with the 3’UTR of Irf2bp2 confirms loss of Irf2bp2. **(B)** Northern blot was stripped and re-probed with 18S RNA to control for loading. **(C–E)** Volcano plots show preponderance of upregulated genes in Irf2bp2-null heart, muscle, and liver tissues. **(F–J)** Venn diagrams show overlap of: **(F)** differentially expressed genes, **(G)** upregulated and **(H)** down-regulated genes in 3 tissues. Ingenuity^®^ pathway analysis identified **(I)** activated and **(J)** inhibited transcription factors common to different Irf2bp2-deficient tissues. Also see **Supplementary Figures 1**–**3** for heatmaps and supplemental Tables 1-7 for gene lists.

Since viable adult Irf2bp2-null mice have not been obtained previously (3), we carried out RNA profiling of adult liver, heart, and skeletal muscle to reveal shared and tissue-specific Irf2bp2-dependent gene programs. Northern blot analysis of total RNA isolated from 3 male Irf2bp2-null and 3 male age-matched wild type mice confirmed loss of Irf2bp2 mRNA in these tissues (**Figures 2A, B**). Two major transcripts were detected with the 3 kilobase (kb) cDNA probe encompassing the entire 3’untranslated sequence of the mouse Irf2bp2 mRNA, one at ~5 kb representing the full length cDNA and another at ~3 kb representing the use of a proximal alternative polyadenylation signal (see the alternative polyadenylation database, http://tools.genxpro.net/apadb/ for details). Note that liver expresses predominantly the shorter transcript, whereas heart and skeletal muscle express both and the longer transcript is ~6-10 times less abundant than the shorter transcript. An aliquot of these RNA samples was used to probe cDNA microarrays to identify differentially expressed genes (**Figures 2C–H** and **Supplementary Table S1**, ArrayExpress accession E-MTAB-11558).

In all 3 tissues, volcano plots (**Figures 2C–E**) revealed that a preponderance of genes was upregulated in Irf2bp2 null mice, reflecting the co-repressor function of Irf2bp2. Venn diagrams also compared the distribution of genes differentially expressed between Irfb2bp2 null and wild type mice in each of the 3 tissues (**Figures 2F–H**, **Supplementary Table S1**). In keeping with the repressor function of Irf2bp2, pathway analysis of transcription regulation indicated that more transcription factors are activated (**Figure 2I**) than inhibited (**Figure 2J**) by Irf2bp2 ablation. Nearly all activated genes (**Supplemental Table S1**) were interferon responsive (http://www.interferome.org/). Consistent with loss of Irf2 repression function, increased Irf1 and Irf3 activity was among the transcription mechanisms activated by Irf2bp2 ablation in all 3 tissues (**Figure 2I**). It should be noted that we did not observe a change in the expression of other Irf2bp-family members (Irf2bp1 and Irf2bpl), in contrast to what was reported for the gene-trap mutant mice (3).

Other transcription regulatory mechanisms activated as a consequence of Irf2bp2 ablation in all 3 tissues (**Figure 2I**) were Spi1 (aka hematopoietic transcription factor PU.1), the NFkB subunits Nfkb1, Nfkbia, Rela, and the Rela-interacting protein myotrophin MTPN (20), the Class II Major Histocompatibility Complex Transactivator Ciita, the nuclear factor of activated T cells Nfat5, the basic helix-loop-helix (bHLH) and PAS-domain proteins Arnt2 and Sim1. Contrary to the reported co-activator function for Irf2bp2 with the glucocorticoid receptor (6), the classic glucocorticoid-inducible metallothionein genes (21) Mt1 and Mt2 we upregulated in all 3 Irf2bp2-null tissues (**Supplemental Table S1**), suggesting instead a co-repressor function of Irf2bp2 with the glucocorticoid receptor.

Transcription factors activated in both liver and muscle include Irf8, Jun, Egr1, Cebpa, Foxl2, the hypoxia-inducible factor Hif1a, and Runx1, a key regulator in hematological malignancies (22). Transcription factors activated in both liver and heart include Irf5 and Irf7, the chromatin remodeling factors Smarca4 and Hmgb1, the signal transducers of activated T cells STAT1 and STAT4, the TGF beta receptor signaling transcription factors Smad3 and Smad4, Ets2, ATF2 (a Jun partner), and the bHLH-PAS protein EPAS1 (aka, Hypoxia-inducible factor 2a). All these factors are likely co-repressed by Irf2bp2. Surprisingly, in the Irf2bp2-null liver, many transcription factors of hematopoietic stem cells (23), including Etv6, Fli1, Ikzf1, and Runx1 were elevated, perhaps reflecting a compensatory mechanism to promote hematopoeisis in the absence of Irfb2p2.

Among transcription factors whose activity was reduced in all 3 tissues by Irf2bp2 ablation were the repressors Sirt1 and Gfi1. Between liver and muscle, activity of the E3 ubiquitin ligase Cbl was reduced, as were Trim24, Bcl6, Nkx2-3, Taf4, Ikzf2 and Prdm16 in liver and heart. Intriguingly, many of the factors that show reduced activity in Irf2bp2 null mice are known to be transcription repressors themselves.

**Figure 3:**
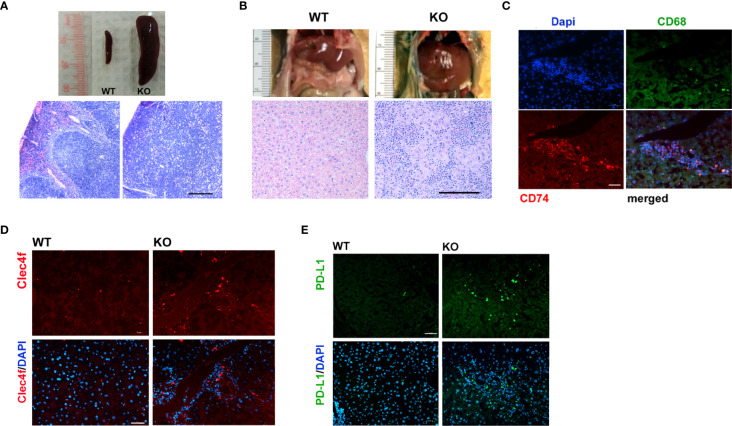
Lymphoma is a prevalent feature of Irf2bp2-null mice. **(A)** Enlarged spleen (splenomegaly) and **(B)** liver (hepatomegaly) often encountered in older (>6 months, n=8) Irf2bp2-null mice were indicative of lymphoma, confirmed in H&E-stained sections. Scale bar, 200 µm. **(C)** Immunofluorescence revealed Cd74-positive infiltrating lymphocytes are distinct from Cd68-postive myeloid cells in Irf2bp2-null liver. **(D)** Irf2bp2-null liver showed increased numbers of Cd68-positive myeloid cells, many positive for the Kupffer cell-specific marker Clec4f. **(E)** Elevated PD-L1 expression was also detected in Irf2bp2-null liver. **(C–E)** Scale bars, 50 µm.

Lymphoma was a prevalent phenotype of adult Irf2bp2-null mice over the age of 6 months in both male and female mice; upon sacrifice, mice displayed an enlarged spleen (**Figure 3A**) and liver (**Figure 3B**). Histological examination revealed disorganized cytoarchitecture in the spleen, with a complete displacement of red pulp (venous sinus with red blood cells) by lymphocyte-rich white pulp, consistent with lymphoma (**Figure 3A**). The livers of Irf2bp2 null mice also showed extensive lymphocytic infiltration (**Figure 3B**) and this was associated with upregulation of many lymphocyte-specific genes (including Cd74, Lsp1, Bcl2a1a, Bcl2a1c, Bcl2a1d, etc.) (**Supplementary Table S1**). Immunofluorescence using Cd74 antibody confirmed lymphoid infiltration in the liver tissue (**Figure 3C**). Lymphocytic infiltration was even detected within the ventricular myocardium (**Supplementary Figure 6**). We also observed elevated expression of myeloid-specific genes Cd68 and Clec4f in Irf2bp2 null livers (**Supplementary Table S1**), and immunofluorescence confirmed a higher number of Cd68 and Clec4f-positive Kupffer cells (**Figure 3D**).

Our microarray data revealed a 3.5-fold increase in PD-L1 (Cd274) expression in Irf2bp2-deficient livers and 1.7-fold increase in the Irf2bp2-deficient heart (**Supplementary Table S1**). PD-L1 expression is known to be suppressed by Irf2bp2 (24, 25). PD-L1 encodes a ligand for PD-1 (aka Pdcd1), a receptor on T lymphocytes that suppresses cancer growth (26). PD-L1 inhibits PD-1 function and is permissive to cancer growth. A marked increase in PD-L1-immunopositive cells was detected in Irf2bp2 null livers (**Figure 3E**), consistent with our array data. Elevated PD-L1 expression is documented in B cell lymphoma (27), and elevated PD-L1 expression likely facilitates lymphoma proliferation in these tissues.

**Figure 4:**
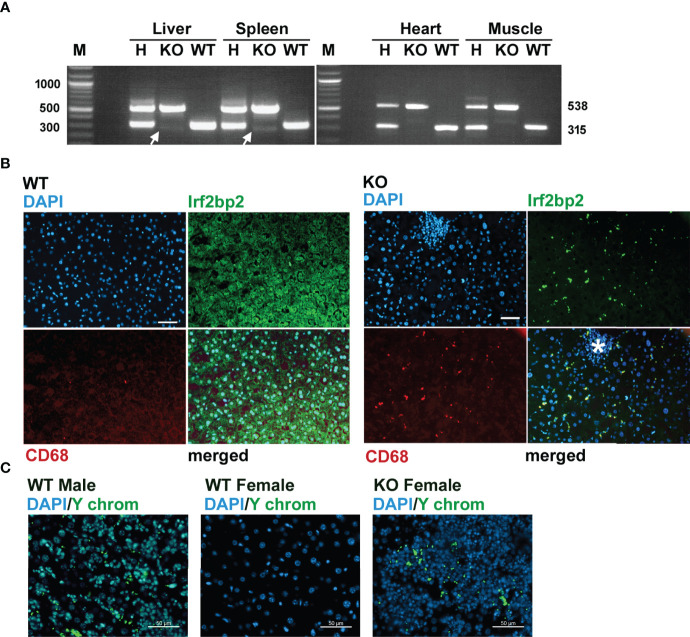
Evidence for erythromyeloid microchimerism in viable Irf2bp2-null mice. **(A)** PCR genotyping of genomic DNA reveals traces of the wild type allele in liver and spleen, but not in heart or skeletal muscle. **(B)** Presence of Irf2bp2-positive and CD68-positive macrophages in Irf2bp2-null liver suggests exogenous origin of erythromyeloid progenitors. Note lymphoid cluster (asterisk) in Irf2bp2-null liver is Irf2bp2-negative. **(C)** *In situ* hybridization reveals Y-chromosome-containing cells in female Irf2bp2-null mice. Scale bars, 50 µm.

Since Irf2bp2 null embryos are detected in the first litter but none survive to term, this result indicates germline ablation of Irf2bp2 is lethal during fetal development. An important question is why a few Irf2bp2-null mice from the 2^nd^ and 3^rd^ litters survive postnatally. PCR genotyping had revealed trace amounts of the wild type allele in genomic DNA from ear biopsies of these Irf2bp2-null mice (**Figure 1B**). Initially, we thought this might be a contaminant since the northern blot (**Figure 2A**) and microarray data (**Supplementary Table S1**) clearly showed loss of Irf2bp2 mRNA in the 3 tissues. However, further comparison of the microarray data revealed that the residual Irf2bp2 signal in the liver of Irf2bp2 null mice was higher than the signal in the heart or skeletal muscle for the same mice. Intrigued by these apparent contradictory data, we carried out further PCR genotyping of DNA isolated from these 3 tissues in Irf2bp2 null mice. While all tissues revealed the floxed Irf2bp2 PCR product, trace levels of the wild type Irf2bp2 allele were detected in DNA from the liver and spleen, but not in skeletal muscle or heart of Irf2bp2-null mice (**Figure 4A**).

Immunofluorescence confirmed the presence of a few Irf2bp2-positive cells in the liver of Irf2bp2-null mice compared to all cells being Irf2bp2-positive in wild type mice (**Figure 4B**). Importantly, these Irf2bp2-positive cells detected in Irf2bp2 null livers were all Cd68-positive myeloid cells (**Figure 4B**). It is also noteworthy that the cluster of lymphocytes (infiltrating lymphoma, **Figure 4B**, asterisk) in the Irf2bp2 null liver was Irf2bp2 negative. The presence of Irf2bp2-positive myeloid cells in the liver of Irf2bp2-null mice suggests that these cells are of exogenous origin.

Microchimerism is a condition whereby embryos acquire from their mother or from prior siblings, exogenous progenitor cells that persist into adulthood (28). If the Irf2bp2-positive myeloid cells in Irf2bp2 null livers were of maternal origin, we should have detected female-specific immune transcripts in the RNA profiles of the 3 male Irf2bp2-null mice: the X-chromosome inactivation transcript Xist, Rsad2 and Oas3 (29). However, none of these transcripts was significantly elevated in male Irf2bp2 null livers compared to WT (Xist, BH = 0.987, KO/WT = 1.01; Oas3, BH = 0.191, KO/WT = 1.256; Rsad2, BH = 0.0874, KO/WT = 1.59). It’s important to point out that the microarrays detected elevated expression of the myeloid-specific transcripts CD68 and Clec4f in the livers of Irf2bp2 null mice (**Supplementary Table S1**) and immunofluorescence confirmed that these markers were only detected in cells that are also Irf2bp2-positive (**Figure 4B**). Therefore, if these Irf2bp2-positive cells were of maternal origin, the Xist RNA should also have been detected. In line with this observation, we were also unable to detect Barr bodies (inactivated X chromosomes) in the nuclei of myeloid cells in the livers of Irf2bp2-deficient male mice, while Barr bodies were readily detectable in female livers (**Supplementary Figure S7**). Thus, given that maternal myeloid-specific transcripts and inactivated X chromosomes were not detected in male Irf2bp2 null livers, the Irf2bp2-positive myeloid cells are unlikely to be of maternal origin, but rather these are likely erythromyeloid progenitor cells left behind from male siblings of prior litters. Indeed, *in situ* hybridization confirmed the presence of cells bearing the male Y chromosome in liver tissue of 3 female Irf2bp2-null mice (**Figure 4C**).

## Discussion

Here, we showed that germline ablation of Irf2bp2 causes fetal lethality in mice, since no viable Irf2bp2 null mice were detected in 1^st^ litters. The presence of a few viable Irf2bp2-null mice in 2^nd^ and 3^rd^ litters carrying Irf2bp2-positive myeloid cells in their livers suggests that these mice are microchimeric, acquiring cells from siblings of prior litters that likely rescue Irf2bp2-deficient mice from fetal lethality. However, since Irf2bp2-positive lymphoblasts are not detected in Irf2bp2-null mice, these chimeric mice are not protected from adult-onset lymphoma caused by loss of Irf2bp2.

In humans, microchimerism (the contribution of exogenously acquired stem cells to the developing fetus) is well known to persist into adulthood (28). The effect of microchimerism can be beneficial in some instances. For example, hemophilia A patients lacking blood clotting factor viii develop alloantibodies to this protein after repeated transfusions. However, rare cases exist where hemophilia A patients do not develop these antibodies because they acquire maternal cells that produce factor viii and induce tolerance (30). While microchimerism may induce tolerance to exogenous factor viii, hemophilia A is not considered life-threatening. Since viable Irf2bp2-null chimeric mice were only found in 2^nd^ and 3^rd^ litters, and because we were unable to detect maternal transcripts or inactivated X chromosomes (Barr bodies) but did find Y chromosome in all female KO livers tested, we propose that sibling microchimerism may account for these cells.

Sibling chimerism has been reported in human dizygotic twins to account for chimeric ABO blood groups (31). Moreover, transmaternal microchimerism as we describe here has been documented in humans where male stem cells transferred from older brothers to their younger sisters were detected in umbilical cord blood samples (32). Remarkably, half of the daughters with older brothers were microchimeric with male cells. However, we could find no report on microchimerism rescuing fetal lethality in humans or mice. To our knowledge, our finding of sibling microchimerism in sequential litters of Irf2bp2 null mice would be the first example of microchimerism rescuing fetal lethality.

Lethality from targeted truncation of Irf2bp2 has been ascribed to a deficit in fetal erythropoiesis, a process that takes place largely in the liver (3). Our observation of empty cranial vessels lacking erythrocytes in an Irf2bp2-null fetus is consistent with this mechanism. The proliferation of erythromyeloid cells in the fetal liver is critical for erythropoiesis during late fetal development (9). The presence of Y-chromosome-containing cells in the livers of viable female Irf2bp2-null mice suggests these cells may be critical to rescue fetal erythropoiesis. The absence of Barr bodies in similar cells of male mice suggests a preferential transfer of male progenitor cells. Unfortunately, because antigen retrieval for Irf2bp2 immunofluorescence was incompatible with *in situ* hybridization of the Y chromosome probe, this technical limitation prevented us from showing that Y-chromosome positive cells were also Irf2bp2-positive. However, given that the number of Y-chromosome-positive and Irf2bp2-positive cells is similar in female Irf2bp2-null mice and that Y-chromosome containing cells were not detected in wild type female livers, these observations suggest they are the same cells. About one third of the expected number of Irf2bp2-null mice was detected in 2^nd^ and 3^rd^ litters, suggesting that transmaternal microchimerism may not always occur, as documented in humans (32), or that sibling-derived myeloid stem cells may not always be acquired in sufficient numbers to rescue Irf2bp2-null mice. Definitive studies to address the frequency and numbers of cells transferred by microchimersim could be addressed with the Irf2bp2-deficient mice generated by the gene trap expressing a β-galactosidase reporter (3), but these studies are beyond the scope of the present manuscript. Chimeric Irf2bp2-null mice that survived to adulthood had a relatively normal hematocrit suggesting their myeloid chimerism may have been adequate to support erythropoiesis through adulthood.

We previously discovered Irf2bp2 as a positive coactivator of the transcription cofactor Vgll4 (5). A recent study in zebrafish reported that Vgll4b is part of a Hif1a/Irf2bp2 complex essential for erythropoiesis (33). Paradoxically, transcription factors enriched in hematopoietic stem cells including Etv6, Fli1, Runx1, and Spi1 were upregulated in tissues of viable Irf2bp2-deficient adult mice, as were several genes tied to erythropoiesis including the oncostatin M receptor. Whether this reflects a compensatory activation due to loss of Irf2bp2 remains unclear. Alternatively, the presence of Irf2bp2-positive chimeric myeloid cells might contribute to upregulation of these erythropoietic factors. We reported that Irf2bp2 protein levels are elevated in response to ischemia in skeletal and cardiac muscle (5), and this would be consistent with stimulated erythropoiesis in chimeric Irf2bp2-null mice.

Those 47 genes that were upregulated in the heart, skeletal muscle and liver tissues, suggest a common Irf2bp2-dependent regulatory mechanism for these genes in these 3 tissues. Conversely, many other genes showed tissue-specific changes in their expression with Irf2bp2 ablation suggesting Irf2bp2 affects transcription differently in different tissues. For example, Irf2bp2 promotes expression of anti-inflammatory genes in macrophages (7). Irf2bp2 is down regulated by lipopolysaccharides (LPS) and ablation of Irf2bp2 in macrophages activates inflammation, impairs macrophage cholesterol efflux and worsens atherosclerosis (7). Ablation of Irf2bp2 in microglia hinders recovery from focal ischemic brain injury (34, 35) and blocks the anxiety-reducing effect of enhanced perinatal maternal care (36). In the heart, Irf2bp2 functions differently. LPS elevates cardiac Irf2pb2 protein levels and Irf2bp2 overexpression in the heart protects against LPS-induced contractile dysfunction (37). In addition, Irf2bp2 protects the heart from hypertrophic stimuli (38) likely by repression of the hypertrophic transcription factor NFAT1 (aka Nfatc2) (4). It is noteworthy that our array data confirmed that Nfatc2-dependent gene expression was significantly activated in the heart of Irf2bp2 null mice.

The presence of lymphoma in Irf2bp2-deficient/chimeric mice was noteworthy because it suggests that loss of Irf2bp2 facilitates this process. It is important to point out that the erythromyeloid lineage does not give rise to lymphocytes (9), so that lymphoid progenitors in chimeric Irf2bp2-null mice should remain Irf2bp2-deficient. Diverse viral pathogens are tied to lymphomagenesis including Epstein Barr virus in Burkitt’s lymphoma, Kaposi’s sarcoma virus, among others. Lymphoma in mice can arise from spontaneous activation of endogenous retroviral sequences (39). Adult T-cell leukemia/lymphoma in humans is caused by the HTLV-1 (human T-cell leukemia virus) retrovirus and is often tied to mutations in Irf2bp2 (40). Whether lymphomagenesis is a direct consequence of Irf2bp2 loss of function or whether Irf2bp2 deficiency increases susceptibility to virus-induced tumors remains to be determined. Our mouse model should enable studies of these mechanisms of lymphomagenesis.

Our finding suggesting that sibling microchimerism in mice can rescue the fetal lethality caused by failure of adequate hepatic erythropoiesis due to Irf2bp2 ablation is potentially clinically important. Given that sibling microchimerism has also been described in humans (31, 32), it will be important to test whether human carriers of autosomal dominant mutations in IRF2BP2 that develop common variable immunodeficiency disorder (11, 12) carry otherwise healthy myeloid cells from older healthy siblings that overcome fetal lethality.

## Methods

### Generation and Genotyping of Irf2bp2-Null Mice

All procedures in mice were approved by the University of Ottawa Animal Care and Use Committee in accordance with the guidelines of the Canadian Council on Animal Care. C57B6-Irf2bp2-flox mice were produced at the University of Connecticut transgenic core facility and mated to Hprt1-Cre mice (19) to enable germline deletion of the entire Irf2bp2 gene (see **Figure 1A**). PCR genotyping used primers to Irf2bp2 sequence flanking the LoxP/Frt site 5’ to the neomycin cassette, and to 3’ sequences flanking the 3’ loxP site, FRT gene target forward (FrtgtF), 5’-TAACCTTGACTCTTGGACAGC-3’, LoxP gene target reverse (LoxgtR), 5’-CCCTTCATGTAAGTATTCTCACTAGG-3’, and LoxP gene target Forward (LoxgtF), 5’-GTGCTCCTTAAGTGTTGCAG-3’. Hemizygous mice were interbred for several consecutive litters and progeny were genotyped by PCR of genomic DNA.

### Timed Pregnancies

To obtain fetuses, timed matings were carried out by intercrossing *Irf2bp2^+/-^* mice and designating 0.5 dpc (days post coitum) to the day of identification of a vaginal plug. Fetuses were harvested from the first litter of a single pregnant female sacrificed by CO_2_ euthanasia at each time point at 11.5, 13.5, 15.5, 16.5 and 18.5 dpc. Fetuses were separated from the placenta and yolk sac, and tails were removed for genotyping.

### RNA Isolation

Total RNA was isolated from WT and Irf2bp2*^-/-^* mouse heart, skeletal muscle, and liver (n=3 males per genotype) using the RNeasy Midi Kit (#75142, Qiagen) for Northern blot and microarray analysis. RNA concentrations and A260/230 purity ratios were measured with the Nanodrop™ 2000 (Model #ND-2000, Thermo Scientific).

### Northern Blot

Total RNA (15 µg/lane) was fractionated on a 3-(N-morpholino)-propanesulfonic acid (MOPS) buffered formaldehyde agarose gel at 80V for 3 hours, as we recently described (41). The gel was washed 4 times in diH_2_O, soaked for 15 minutes in water containing 50 μL ethidium bromide (10 mg/mL), then washed again 6 times in diH_2_O and photographed under UV light to visualize the 18S and 28S RNAs. RNA was transferred to nylon membrane (GeneScreen Plus Hybridization Transfer Membrane, #NEF988, Perkin Elmer) by overnight capillary transfer in 10X SSPE solution (1.5 M NaCl, 100 mM NaH_2_PO_4_, 100 mM Na_2_EDTA). RNA was immobilized by UV crosslinking and membrane was prehybridized in ExpressHyb™ Hybridization Solution (#636832, Clontech) at 65°C for 1 hour.

The Irf2bp2 3’UTR cDNA probe was released by SalI/NotI digestion (clone ID #6476448, Integrated DNA Technologies) purified from an agarose gel. 50 pmol of probe was denatured, random primed with Klenow fragment using the Random Primers Labeling Kit (#18187013, Life Technologies Inc), and radioactively labeled with [α-^32^P]dCTP (#BLU013H250UC, Perkin Elmer Health Sciences). A cDNA probe for 18S RNA (control) was radioactively end-labeled with [γ-^32^P]-ATP (#BLU502Z250UC, Perkin Elmer Health Sciences) in a T4 Polynucleotide Kinase (#M0201S, New England Biolabs) reaction. Radioactive probes were added to 10 mL of fresh hybridization solution and incubated with the pre-hybridized membrane overnight at 65°C. The next day, the membrane was transferred to a tray and washed 3 times with 0.2% SDS in 2X SSPE buffer. The membrane was then washed 2 times in 0.2% SDS in 0.2X SSPE buffer in a 50°C bath for 15 minutes each. The membrane was covered in plastic wrap and exposed to a storage Phosphor Screen overnight and image was developed on a STORM 860 phosphorimager (Amersham Biosciences).

### Preparation of cDNA for Microarray Analysis

Microarray analysis was performed using Mouse Gene 1.0 ST Arrays (Affymetrix). Total RNA was isolated and purified from mouse heart, skeletal muscle, and liver as described previously, and samples were sent to the Affymetrix Microarray Facility at StemCore Laboratories (Ottawa Hospital Research Institute). RNA integrity was assessed using the Agilent 2100 Bioanalyzer (#G2943CA, Agilent Technologies), which provides an RNA integrity number (RIN) from microfluidics analysis. The Ambion Whole-Transcript Expression kit was used in conjunction with the Affymetrix GeneChip Whole-Transcript Terminal Labeling and Controls Reagent kit to prepare samples for the Mouse Gene 1.0 ST Array.

RNA for microarray analysis was prepared by generating sense-strand cDNA for fragmentation and labeling with the Ambion Whole-Transcript Expression Kit (#4411974, Life Technologies). First-strand cDNA was synthesized by reverse-transcription from total RNA and second-strand cDNA was synthesized using DNA polymerase and RNase H simultaneously to degrade the RNA. Antisense cRNA was synthesized and amplified by *in vitro* transcription of the second-strand cDNA template using T7 RNA polymerase. cRNA was purified using Nucleic Acid Binding Beads, washed twice with Nucleic Acid Wash Solution, and eluted with 55°C preheated Elution Solution. The cRNA was briefly placed on ice and its yield was assessed by UV absorbance at 260 nm. Next, 10 μg cRNA was mixed with random primers to synthesize 2nd-cycle cDNA by reverse transcription. The cRNA template was degraded with RNase H, and the cDNA was purified to remove enzymes, salts, and unincorporated dNTPs.

### Histology and Immunofluorescence Microscopy

Tissues harvested for H&E staining were fixed in 4% paraformaldehyde (PFA) in PBS for 24-48 hours, then transferred to 70% ethanol. Samples were paraffin-embedded, sectioned at 5 μm thickness, then H&E stained by the University of Ottawa Department of Pathology and Laboratory Medicine. For immunofluorescence, samples were fixed in 4% PFA, dehydrated in 30% sucrose overnight and frozen 10 µm sections were processed for immunofluorescence. Antigen retrieval was carried out in citrate buffer (10 mM Citric Acid, 0.05% Tween 20, pH 6.0) at 95°C for 20 minutes. Sections were blocked in 10% normal serum of the species used for secondary antibody. Antibodies were as follows: mouse monoclonal to the macrophage-specific antigen CD68 (mab101141 R&D Systems Inc.) was revealed with a chicken anti-rabbit IgG antibody conjugated to Alexa Fluor 488 (#A-21441, ThermoFisher Inc.), the PD-L1 (CD274) antibody was a rabbit polyclonal (SAB4301882, Sigma Aldrich Inc.), the lymphocyte-specific antigen CD74 was a mouse monoclonal conjugated to Alexa Fluor 647 (FAB35901R, R&D Systems Inc., Minneapolis, MN), the Kupffer cell specific antigen Clec4f (mab2784, 1:200, R&D Systems Inc., Minneapolis, MN), Barr bodies were revealed by an anti-ubiquityl-Histone H2A antibody, as described (42), clone E6C5 (Sigma Aldrich Inc.) revealed with a goat anti mouse IgG,IgM(H+L) secondary antibody conjugated to Alexa Fluor 488 (A-10680, ThermoFisher Inc). The rabbit anti-Irf2bp2 antibody was described previously (5). Nuclei were revealed with DAPI and sections were treated with Vector^®^ True VIEW^®^ Autofluorescence Quenching Kit to minimize autofluorescence (VECTSP8400, Vectorlabs Inc.) and mounted with coverslips in VECTASHIELD^®^ Antifade Mounting Medium (VECTH1000, Vectorlabs Inc.). Images were acquired on a Zeiss M1 microscope.

### *In Situ* Hybridization

Paraffin sections from liver tissue isolated from adult mice were probed with a Y chromosome-specific fluorescent probe (Creative Bioarray) and counterstained with DAPI. The probe was incubated overnight at 37 C for 16 hours, followed by autofluorescence quenching for 5 minutes (Vector Laboratories Vector TrueVIEW Autofluorescence Quenching Kit #SP-8400), then stained with DAPI for 10 minutes and mounted with a coverslip in VECTASHIELD mounting medium.

### Ingenuity Pathway Analysis

Differentially expressed genes identified by array analysis were further compared by Ingenuity^®^ pathway analysis (Qiagen) to identify upstream transcription factors whose target genes are upregulated or downregulated by Irf2bp2 ablation.

### Statistical Analysis

The Robust Multi-array Average (RMA) algorithm was used to transform and normalize the raw intensity values of the probes using Affymetrix Expression Console™ Software. To identify differential gene expression between wildtype and knockout tissues, a Bayesian t-test analysis was performed using the Cyber-T analysis package for R (43). The Benjamini-Hochberg (BH) False Discovery Rate (FDR) method was used to correct for multiple testing and the significance threshold was set at 0.05, equal to an FDR of 5% (44). Probes that were not linked to a gene through annotation were excluded from analysis.

## Data Availability Statement

The datasets presented in this study can be found in online repositories. The names of the repository/repositories and accession number(s) can be found below: ArrayExpress accession E-MTAB-11558.

## Ethics Statement

The animal study was reviewed and approved by University of Ottawa Animal Care and Use Committee.

## Author Contributions

RV, TH, AD, NH, KK, and FS obtained and analyzed the data, RV, H-HC, and AS wrote the manuscript, and H-HC and AS obtained research funding. All authors contributed to the article and approved the submitted version.

## Funding

AS and H-HC are supported by operating grants from the Canadian Institutes of Health Research (376403, H-HC; 376503, AS), Discovery grants from the Natural Sciences and Engineering Research Council of Canada (RGPIN-2019-03942, H-HC; RGPIN-2016-04985, AS), a grant from the Canadian Diabetes Association (OG-3-14-4567-HC, HHC & AFRS), grants-in-aid by the Heart and Stroke Foundation of Canada (G-16-00014085, AS; G-18-0022157, HHC) and a midcareer salary award by the Heart and Stroke Foundation of Ontario (H-HC). RV was supported by a graduate student scholarship of the University of Ottawa Heart Institute.

## Conflict of Interest

The authors declare that the research was conducted in the absence of any commercial or financial relationships that could be construed as a potential conflict of interest.

## Publisher’s Note

All claims expressed in this article are solely those of the authors and do not necessarily represent those of their affiliated organizations, or those of the publisher, the editors and the reviewers. Any product that may be evaluated in this article, or claim that may be made by its manufacturer, is not guaranteed or endorsed by the publisher.

## Supplementary Material

Supplementary Figure 1 Heat map of differentially expressed genes in liver of Irf2bp2 null mice compared to WT controls.

Supplementary Figure 2 Heat map of differentially expressed genes in skeletal muscle of Irf2bp2 null mice compared to WT controls.

Supplementary Figure 3 Heat map of differentially expressed genes in heart of Irf2bp2 null mice compared to WT controls.

Supplementary Figure 4 Hematology profile from an adult Irf2bp2-null mouse.

Supplementary Figure 5 Hematology profile from an adult hemizygous Irf2bp2 mouse.

Supplementary Figure 6 Lymphocyte infiltration into the ventricular myocardium of an Irf2bp2-null mouse with lymphoma.

Supplementary Figure 7 Absence of Barr bodies in liver of Irf2bp2-null male mouse.

Supplementary Table 1 Lists of differentially expressed genes in Irf2bp2-null mice.
