# Supplementary figures and images for: Savior Siblings Might Rescue Fetal Lethality But Not Adult Lymphoma in Irf2bp2-Null Mice
Source: Front Immunol. 2022 Jul 4;13:868053. doi: 10.3389/fimmu.2022.868053 (PMC9295810; doi:10.3389/fimmu.2022.868053)

Color Key

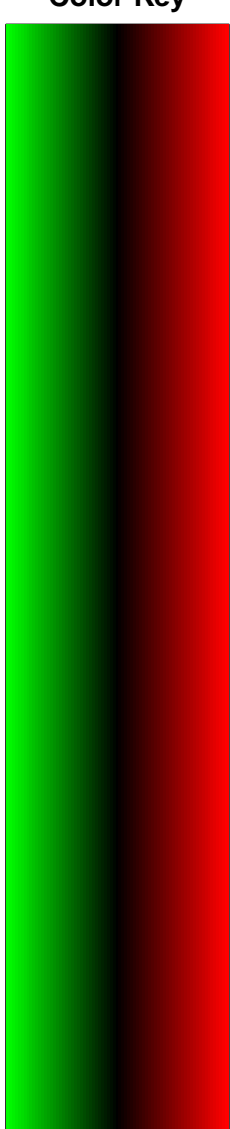

Row Z-Score

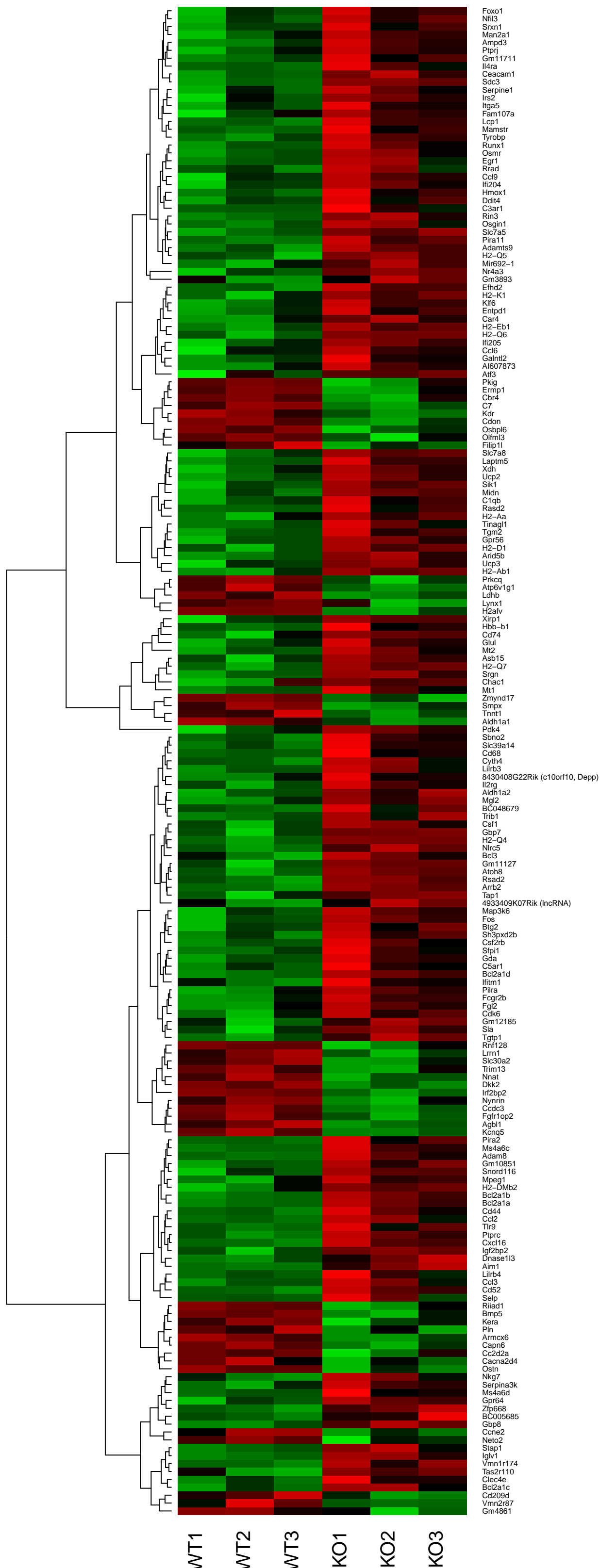

Supplement: Supplementary Figure 2 — Heat map of differentially expressed genes in skeletal muscle of Irf2bp2 null mice compared to WT controls. [file DataSheet_2.pdf]

Color Key

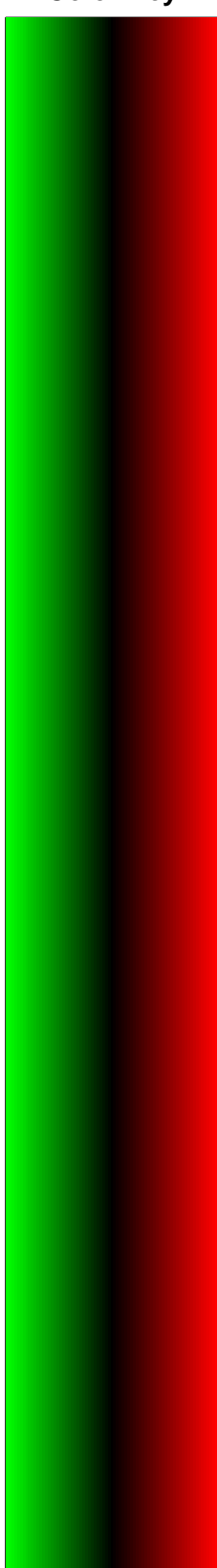

Row Z-Score

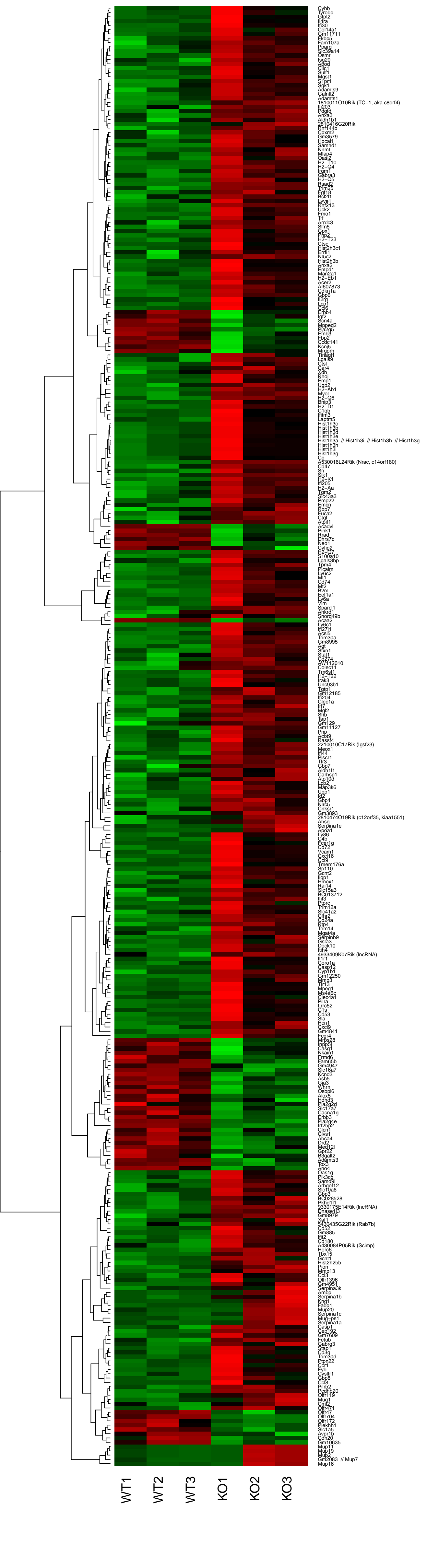

Supplement: Supplementary Figure 3 — Heat map of differentially expressed genes in heart of Irf2bp2 null mice compared to WT controls. [file DataSheet_3.pdf]
