# Supplementary material for: Savior Siblings Might Rescue Fetal Lethality But Not Adult Lymphoma in Irf2bp2-Null Mice
Source: Front Immunol. 2022 Jul 4;13:868053. doi: 10.3389/fimmu.2022.868053 (PMC9295810; doi:10.3389/fimmu.2022.868053)
Supplement: Supplementary Figure 4 — Hematology profile from an adult Irf2bp2-null mouse. [file DataSheet_4.pdf]

LABORATORY REPORT

IDEXX LABORATORIES

1345 Denison Street
Markham, Ontario L3R 5V2
Tel: (416) 798-4988
Toll-free: (800) 667-3411
Fax: (905) 475-7309

**Visit No:**	**V24456305**
**Lab No:**	**4299012**
**File #:**	E44008205833
**Submission Date:**	2012-10-10 04:04:23
**Completed Date:**	2012-10-10 05:47:42

**Clinic:**	E44 University of Ottawa - ACSS	**Fax:**	613-562-5467	**Phone:**	613-562-5800
**Clinician:**		**Fax:**		**Phone:**	
**Patient:**	KEYHANIAN, 167(KO)	**Species:**	Mouse	**Breed:**	
**Sex:**	Male	**Age:**	6 Weeks		

HEMATOLOGY

Test	Results	Reference	Units	Lab
WBC	1.9	-	x10E9/L	TOR
RBC	7.7	-	x10E12/L	TOR
Hemoglobin	114	-	g/L	TOR
Hematocrit	0.39	-	L/L	TOR
MCV	52	-	fl	TOR
MCH	15	-	pg	TOR
MCHC	289	-	g/L	TOR
Reticulocytes	3.5	-	%	TOR
Platelets	472	-	x10E9/L	TOR

**Differential:**	%	abs.		
Bands	0.0	0.0	-	x10E9/L
Neutrophils	8.0	0.2	-	x10E9/L
Lymphocytes	84.0	1.6	-	x10E9/L
Monocytes	8.0	0.2	-	x10E9/L
Eosinophils	0.0	0.0	-	x10E9/L
Basophils	0.0	0.0	-	x10E9/L

Morphology:

WBC MORPHOLOGY
Normal
RBC MORPHOLOGY
Polychromasia 2-5 /HPF
PLT MORPHOLOGY
Normal
COMMENTS
Small clot present

Comment Reference intervals, where reported, were established using internal data.
Please send duplicate results to: email: kian.kayhanian@gmail.com
Small clot present, CBC results may be compromised LB

**Tests requested:**	HCBC
**Tests to follow:**	

(GMT-05:00) Eastern Time (US and Canada)
