# Supplementary material for: Savior Siblings Might Rescue Fetal Lethality But Not Adult Lymphoma in Irf2bp2-Null Mice
Source: Front Immunol. 2022 Jul 4;13:868053. doi: 10.3389/fimmu.2022.868053 (PMC9295810; doi:10.3389/fimmu.2022.868053)
Supplement: Supplementary Figure 5 — Hematology profile from an adult hemizygous Irf2bp2 mouse. [file DataSheet_5.pdf]

LABORATORY REPORT

IDEXX LABORATORIES
1345 Denison Street
Markham, Ontario L3R 5V2
Tel: (416) 798-4988
Toll-free: (800) 667-3411
Fax: (905) 475-7309

**Visit No:**	**V24456307**
**Lab No:**	**4299014**
**File #:**	E44008205839
**Submission Date:**	2012-10-10 04:07:12
**Completed Date:**	2012-10-10 05:48:04

**Clinic:**	E44 University of Ottawa - ACSS	**Fax:**	613-562-5467	**Phone:**	613-562-5800
**Clinician:**		**Fax:**		**Phone:**	
**Patient:**	KEYHANIAN, 154(HEMI)	**Species:**	Mouse	**Breed:**	
**Sex:**	Female	**Age:**	6 Weeks		

HEMATOLOGY

Test	Results	Reference	Units	Lab
WBC	2.2	-	x10E9/L	TOR
RBC	8.8	-	x10E12/L	TOR
Hemoglobin	131	-	g/L	TOR
Hematocrit	0.49	-	L/L	TOR
MCV	56	-	fl	TOR
MCH	15	-	pg	TOR
MCHC	269	-	g/L	TOR
Reticulocytes	2.6	-	%	TOR
Platelets	834	-	x10E9/L	TOR

**Differential:**	%	abs.		
Bands	0.0	0.0	-	x10E9/L
Neutrophils	17.0	0.4	-	x10E9/L
Lymphocytes	79.0	1.7	-	x10E9/L
Monocytes	2.0	0.0	-	x10E9/L
Eosinophils	2.0	0.0	-	x10E9/L
Basophils	0.0	0.0	-	x10E9/L

Morphology:

WBC MORPHOLOGY
Normal
RBC MORPHOLOGY
Polychromasia 2-5 /HPF
PLT MORPHOLOGY
Normal
COMMENTS
Small clot present

Comment Reference intervals, where reported, were established using internal data.
Please send duplicate results to: Email: kian.kayhanian@gmail.com
Small clot present, CBC results may be compromised LB

**Tests requested:**	HCBC
**Tests to follow:**	

(GMT-05:00) Eastern Time (US and Canada)
