# Supplementary material for: Savior Siblings Might Rescue Fetal Lethality But Not Adult Lymphoma in Irf2bp2-Null Mice
Source: Front Immunol. 2022 Jul 4;13:868053. doi: 10.3389/fimmu.2022.868053 (PMC9295810; doi:10.3389/fimmu.2022.868053)
Supplement: Supplementary Figure 6 — Lymphocyte infiltration into the ventricular myocardium of an Irf2bp2-null mouse with lymphoma. [file DataSheet_6.pdf]

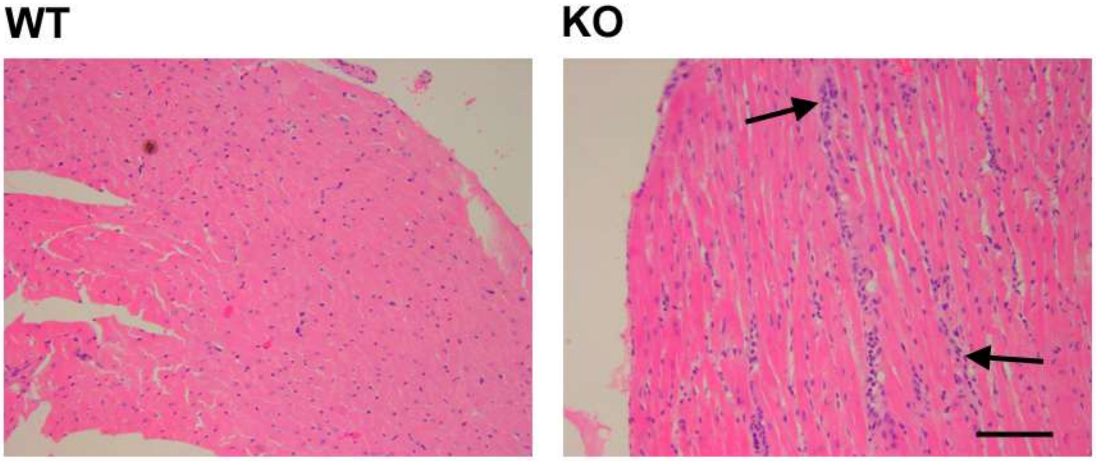

Fig. S6 Extensive lymphocytic infiltration of the myocardium in Irf2bp2 KO mouse with lymphoma. H&E staining reveals clusters of lymphocytes (arrow). Scale bar = µ100 m.
