# Supplementary material for: Savior Siblings Might Rescue Fetal Lethality But Not Adult Lymphoma in Irf2bp2-Null Mice
Source: Front Immunol. 2022 Jul 4;13:868053. doi: 10.3389/fimmu.2022.868053 (PMC9295810; doi:10.3389/fimmu.2022.868053)
Supplement: Supplementary Figure 7 — Absence of Barr bodies in liver of Irf2bp2-null male mouse. [file DataSheet_7.pdf]

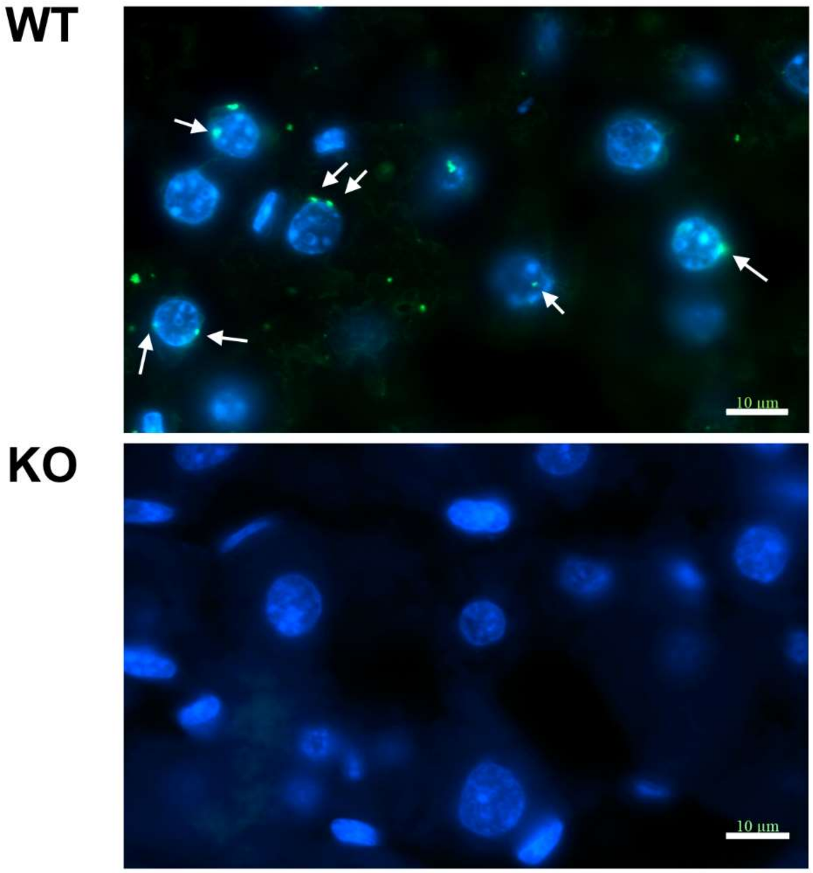

Fig. S7 Absence of Barr bodies in male Irf2bp2KO liver. Barr bodies (arrows) were revealed in female wild type liver by an anti-ubiquityl-Histone H2A antibody, as described (Baarends *et al*, 2005). In contrast, none were detected in any male Irf2bp2KO liver sample, suggesting non-maternal (same-sex sibling) origin of Irf2bp2-positive myeloid cells. Scale bar = 10 µm.
