# Supplementary material for: Savior Siblings Might Rescue Fetal Lethality But Not Adult Lymphoma in Irf2bp2-Null Mice
Source: Front Immunol. 2022 Jul 4;13:868053. doi: 10.3389/fimmu.2022.868053 (PMC9295810; doi:10.3389/fimmu.2022.868053)
Supplement: Supplementary Figure 1 — Heat map of differentially expressed genes in liver of Irf2bp2 null mice compared to WT controls. [file DataSheet_1.pdf]

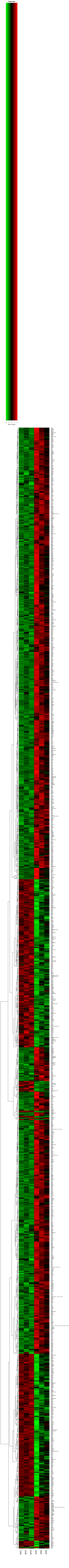

Color Key
−2 −1 0 1 2
Row Z−Score
WT1
WT2
WT3
KO1
KO2
KO3
